# The 3’-Jα Region of the TCRα Locus Bears Gene Regulatory Activity in Thymic and Peripheral T Cells

**DOI:** 10.1371/journal.pone.0132856

**Published:** 2015-07-15

**Authors:** Martina Kučerová-Levisohn, Stefan Knirr, Rosa I. Mejia, Benjamin D. Ortiz

**Affiliations:** 1 Department of Biological Sciences, City University of New York, Hunter College, New York, New York, United States of America; 2 Doctoral Program in Biology, City University of New York, Graduate Center, New York, New York, United States of America; Pohang University of Science and Technology (POSTECH), REPUBLIC OF KOREA

## Abstract

Much progress has been made in understanding the important *cis*-mediated controls on mouse TCRα gene function, including identification of the Eα enhancer and TCRα locus control region (LCR). Nevertheless, previous data have suggested that other *cis*-regulatory elements may reside in the locus outside of the Eα/LCR. Based on prior findings, we hypothesized the existence of gene regulatory elements in a 3.9-kb region 5’ of the Cα exons. Using DNase hypersensitivity assays and TCRα BAC reporter transgenes in mice, we detected gene regulatory activity within this 3.9-kb region. This region is active in both thymic and peripheral T cells, and selectively affects upstream, but not downstream, gene expression. Together, these data indicate the existence of a novel *cis*-acting regulatory complex that contributes to TCRα transgene expression *in vivo*. The active chromatin sites we discovered within this region would remain in the locus after TCRα gene rearrangement, and thus may contribute to endogenous TCRα gene activity, particularly in peripheral T cells, where the Eα element has been found to be inactive.

## Introduction

The functional rearrangement and expression of the TCRα gene during T cell development results in cell surface αß TCR complex emergence. These processes are tightly regulated in *cis* at the TCRα gene locus [1, 2]. DNase I hypersensitive sites (HS) located 3’ of the Cα constant region exons comprise a locus control region (LCR) that supports a great deal of TCRα transcriptional regulatory characteristics [3, 4]. In particular, the region of HS1 (which contains the Eα transcriptional enhancer [5]) and the HS1 prime (HS1’) element [6] is critical for normal TCRα gene rearrangement [7]. This region also very strongly increases transcription levels in T cells *in vivo* [6, 7], likely via epigenetic regulation of chromatin states [8, 9].

While the importance of the HS1/HS1’ DNA region to the function of the TCRα locus is indisputable, limited TCRα gene recombination is observed in its absence. This is accompanied by TCRα transcription levels adequate to support maintenance of a peripheral T cell population with normal αß TCR levels [7]. It has been speculated that the remaining elements of the TCRα LCR might explain this HS1/HS1’ region-independent TCRα gene activity [7]. Nevertheless, heterologous reporter transgenes linked to the TCRα LCR, while mimicking the kinetics/levels of TCRα gene expression in thymocytes [10], are transcribed at lower than expected levels in peripheral T cells [4]. This phenomenon is congruent with prior data indicating that while the Eα/HS1 element is active in thymocytes, its deletion from reporter transgenes had no impact on transgene mRNA levels in peripheral lymphoid organs [6]. Using completely different experimental models, a very recent report similarly concluded that, by multiple criteria, the Eα element is inactive in peripheral T cells [11]. Together these reports strongly suggest the presence of additional TCRα gene regulatory elements in the locus outside of the LCR capable of maintaining transcription of the TCRα gene in peripheral T cells.

In contrast to heterologous, TCRα LCR-driven reporter genes, transgenic mice bearing cognate TCRα transgenes linked to the full TCRα LCR display normal levels of transgenic αß TCR in peripheral T cells [12]. A major difference between the TCRα transgenes and the heterologous TCRα LCR reporter constructs previously analyzed is that the former include TCRα locus DNA sequences upstream of the LCR up to a SacI restriction site located near Jα3 [13]. The vast majority of this DNA region would remain present in the endogenous locus following functional TCRα gene rearrangement. We hypothesized that transcriptional control elements might be present in this DNA region between the Jα3-proximal SacI site and the LCR. In the present study, we examined this region for indications of gene regulatory activity.

We report the presence of an array of DNase I hypersensitive sites (HS) in a region of the mouse TCRα locus that ranges from the Jα2 segment to the Cα1 exon. We previously described a TCRα locus derived bacterial artificial chromosome (BAC) construct containing two reporter genes [14]. One, Vα promoter-driven reporter lies upstream of the HS cluster in the orientation and position of the TCRα gene. The second gene reports the activity of the Dad 1 promoter that lies downstream of both the HS cluster and TCRα LCR. Deletion from this construct of a 3.9-kb region of TCRα locus DNA, that includes the Jα3-proximal SacI site and the identified HS clusters, impairs upstream, but not downstream reporter gene activity in transgenic mice. The deleted region is active in both thymocytes and peripheral T cells. The HS cluster discovered here lies in a region of the locus that would remain in all functionally rearranged TCRα alleles. Therefore, this novel regulatory region may play a role in endogenous TCRα gene activity. It may be especially important to maintaining TCRα mRNA levels in peripheral T cells.

## Materials and Methods

### Ethics Statement

Transgenic animal studies presented in this work have been reviewed and approved by the Hunter College Institutional Animal Care and Use Committee (protocol # BO 10/17-01). Animals are euthanized by carbon dioxide inhalation in conformance with American Veterinary Medical Association recommendations.

### TCRα/Dad1 bacterial artificial chromosome (BAC) dual-reporter constructs

The wild type dual-reporter BAC construct used in this study has been previously described [14]. The mutant BAC was engineered to delete a 3.9-kb region spanning from 38-bp 5’ of a SacI site (located between Jα4 and Jα3) to 9-bp 3’ of an EcoRV site within the Cα constant region exon 1. BAC modifications utilized Red/ET recombination technology (Gene Bridges) [15].

### Transgenic mice

Wild type (76.2-kb) and mutant (72.3-kb) dual-reporter BAC fragments were released from the parent BAC pBACe3.6 vector backbone using NotI and FseI. Transgenic founders bearing intact integrants were identified by Southern blot and PCR screening. Founders were then outcrossed to C57BL/6 mice (Taconic) to establish transgenic mouse lines. The relative transgene copy numbers among the individual mouse lines were determined by multiple Southern blot experiments as described previously [16]. Four independent wild type (line 36, 42, 62 and 71) and four independent mutant reporter BAC transgenic mouse lines (line 4, 18, 25 and 30) are analyzed in this study.

### DNase I Hypersensitivity Assay

These experiments were carried out as previously described [17] using nuclei of MACS (Miltenyi) purified spleen T cells from C57BL/6 mice that were subjected to titrating amounts of DNase I (Worthington). NdeI restriction enzyme digestion was used to generate the 6.7-kb parent fragment of the TCRα locus examined. A 669-bp probe was generated by PCR using the parent BAC clone as a template and the following primers: forward: 5’-atggctgagggaaaggtctacg-3’ and reverse: 5’-agaaaagtctctgggaactggtgtc-3’. The probe was labeled with [α-^32^P] dCTP and/or [α-^32^P] dATP using the RadPrime DNA Labeling System (Life Technologies).

### Flow cytometric analyses

1x10^6^ splenocytes were pretreated in 100 μL of FACS staining medium (RPMI 1640 supplemented with 3% FBS and 10mM HEPES buffer) for 10 min at 4°C with 1μg of rat anti-CD16/32 (Clone 2.4G2, Life Technologies) to block Fc receptors. Afterwards, 0.2-.5 μg of the Abs (from BD Biosciences) were added and incubated for 20 min at 4°C, followed by three washes with FACS staining medium. The Ab clones used were mouse anti-human CD2 (clone S5.2), mouse anti-rat CD2 (clone OX-34), and hamster anti-mouse TCRß chain (clone H57-597). Samples were acquired using a FACSCalibur device (BD Biosciences) and the collected data was analyzed with FlowJo software (Tree Star).

### RNA Analysis

5–10 μg of total thymic RNA was separated by agarose gel electrophoresis and transferred to neutral nylon membrane (Amersham) for Northern blot analyses. Probes for detection of reporter hCD2 and rCD2 transgene RNA were previously described [15]. A highly specific 18S rRNA 20-mer oligonucleotide [18] was used to control for loading and efficiency of transfer. PhoshorImager analyses were used to obtain normalized transgene expression levels (per copy). For quantitative (q)RT-PCR, RNA samples from MACS purified spleen T cells (>91% purity) were isolated using the RNeasy Mini Kit (Qiagen). The QuantiTect Reverse Transcription Kit (Qiagen) was utilized for cDNAs syntheses. Signals from hCD2-specific primers were normalized to endogenous TCRα as previously described [10]. Signals from rCD2-specific primers (fw: 5’-ccagtgccttgttcaggatacg-3’, rev: 5’-ggagtttctttctgctcttcagcc-3’) were normalized to endogenous Dad1 expression levels. For this, a forward primer specific for exon 1 (that is not present in the BAC reporter): (5’-tgcagttcggctactgtctcc-3’) was used with a reverse primer complimentary to Dad1 exon 3 (5’-ggaaagtaagggctacagtgagg-3’). qRT-PCR experiments were carried out using DyNAmo HS SYBR Green qPCR Kit (New England BioLabs) and the ViiA7 system (Applied Biosystems).

## Results

### A region of active chromatin in the 3’-Jα region

**Fig 1:**
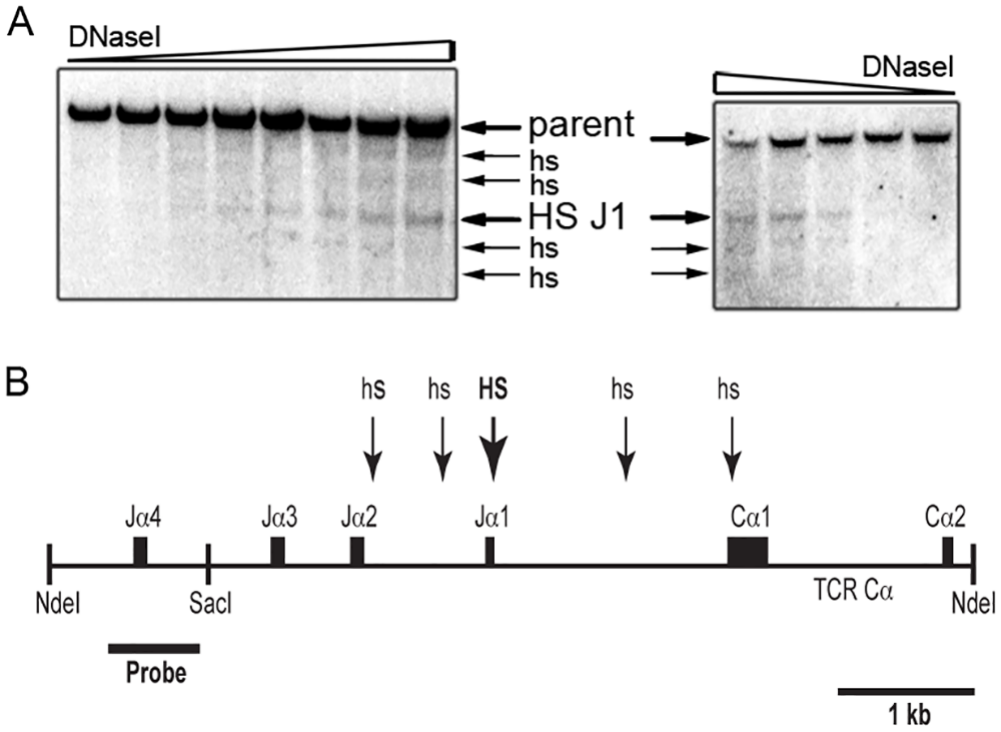
DNase I hypersensitivity sites (HS) within the Jα2 to Cα1 region. **(A**) Spleen T cell nuclei from C57BL/6N mouse were subjected to DNase I titration. Arrows indicate the positions of the 6.7-kb NdeI parent fragment and HS. Results from two independent, representative experiments are shown. **(B)** Scaled diagram of the approximate locations of detected HS (arrows). The thick arrow near Jα1 indicates the most prominent HS.

We used DNase I hypersensitivity assays to examine the chromatin state of a 6.7-kb NdeI fragment of the TCRα locus containing the Jα1 to Jα4 segments and Cα exons 1 and 2 (Fig 1). Of the four Jα segments present in this genomic fragment, only Jα2 supports functional TCRα protein production [19]. To examine this region for HS in a way that minimizes the potential complication of Vα-Jα recombination-mediated production of multiple parent restriction fragments, we selected a probe that would only detect alleles at which Vα-Jα recombination occurred to a Jα segment upstream of Jα3. To minimize the presence of non-functional Jα4 rearranged TCRα alleles in the DNase-treated genomic DNA samples, these assays were carried out using nuclei from isolated peripheral T cells.

A cluster of five HS was detected (Fig 1). The most prominent of these HS is proximal to the Jα1 segment (HS-J1), which is located near the center of the HS array. The other four HS are less prominent. In multiple, independent experiments, these four HS display variable intensity in comparison to each other. Two of these HS approximately localize to DNA in between the Jα2 and Jα1 segments. A third HS is found in the Jα1-Cα1 intron, while the fourth localizes to the Cα1 exon. Since Jα1 is a non-functional pseudo-Jα segment [19], the region containing the HS array detected would remain present in all functionally rearranged TCRα alleles.

### Testing *cis*-acting TCRα gene regulatory activity in transgenic mice

**Fig 2:**
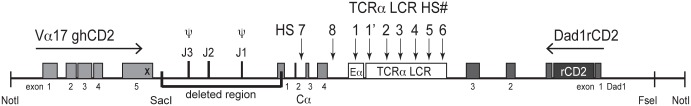
Diagram (not to scale) of the TCRα/Dad1 dual-reporter BAC construct [14]. Horizontal arrows indicate the orientation of the two reporter genes. A Vα17 promoter drives expression of a genomic human CD2 reporter gene (ghCD2). The rat CD2 reporter is driven by the Dad 1 promoter. Vertical arrows indicate the location of the HS of the TCRα LCR (including the TCRα enhancer, Eα). The 3.9-kb region deletion in the mutant BAC is marked and runs from 38-bp 5’ of the SacI site through 30-bp 5’ of the end of Cα exon 1.

We previously described a dual-reporter transgene based on a mouse TCRα/Dad1 locus BAC construct (Fig 2) [14]. At the 5’ end of the BAC, a human CD2 (hCD2) reporter in genomic configuration is integrated in a position upstream of the Cα exons. The hCD2 reporter gene in this construct is driven by a TCRα V-region promoter that was fused in frame with the hCD2 ATG start codon. In addition, a rat CD2 (rCD2) cDNA was fused in frame with the ATG start codon located in Dad1 exon 1. Thus, the rCD2 reporter gene is driven by the Dad1 promoter. To test the function of the above-described target region of the locus, we created a deletion mutant in the context of this dual-reporter BAC that removed a 3.9-kb SacI to EcoRV fragment stretching from upstream of Jα3 to Cα1 (target region). Both wild type and target region mutant BAC constructs were liberated from the vector backbone and used to create transgenic mice.

### Target region deletion impairs upstream, but not downstream reporter gene expression

**Fig 3:**
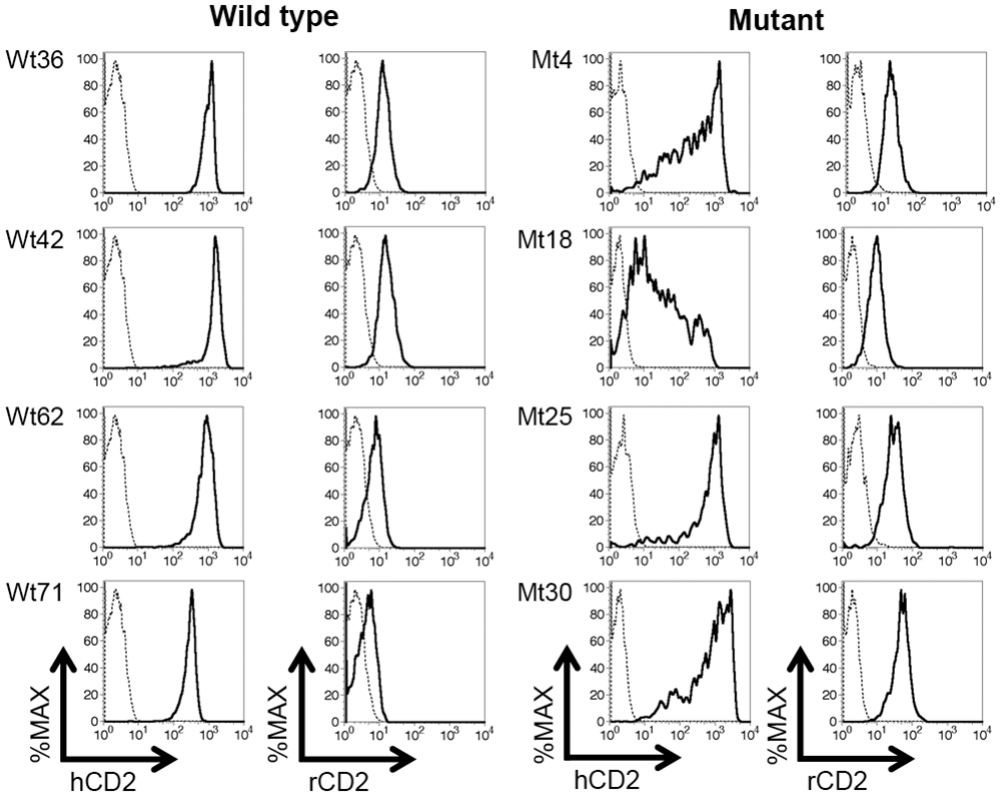
Impaired hCD2 reporter expression in the absence of the deleted region. Flow cytometry analyses of human CD2 (hCD2) and rat CD2 (rCD2) reporter gene expression in spleen T cells (TCRβ^+^) from the indicated, independent wild type (Wt) and mutant (Mt) dual-reporter BAC transgenic mouse lines. Reporter gene expression in transgenic (solid line) and non-transgenic control (dashed line) cells is shown.

Flow cytometry analyses were carried out to initially assess the impact of target region-deletion on BAC reporter gene expression (Fig 3). Four wild type and four mutant reporter BAC transgenic mouse lines were analyzed. T cells from wild type reporter BAC transgenic mice expressed the upstream hCD2 reporter gene at high and relatively uniform levels on a per cell basis. In contrast, per cell hCD2 expression levels in the target region-deletion mutant BAC were more variegated in transgenic T cells. Expression of the downstream, rCD2 reporter gene was detected in all lines at low, but uniform levels in T cells. The deletion mutation did not appear to affect the uniformity of rCD2 expression levels.

**Fig 4:**
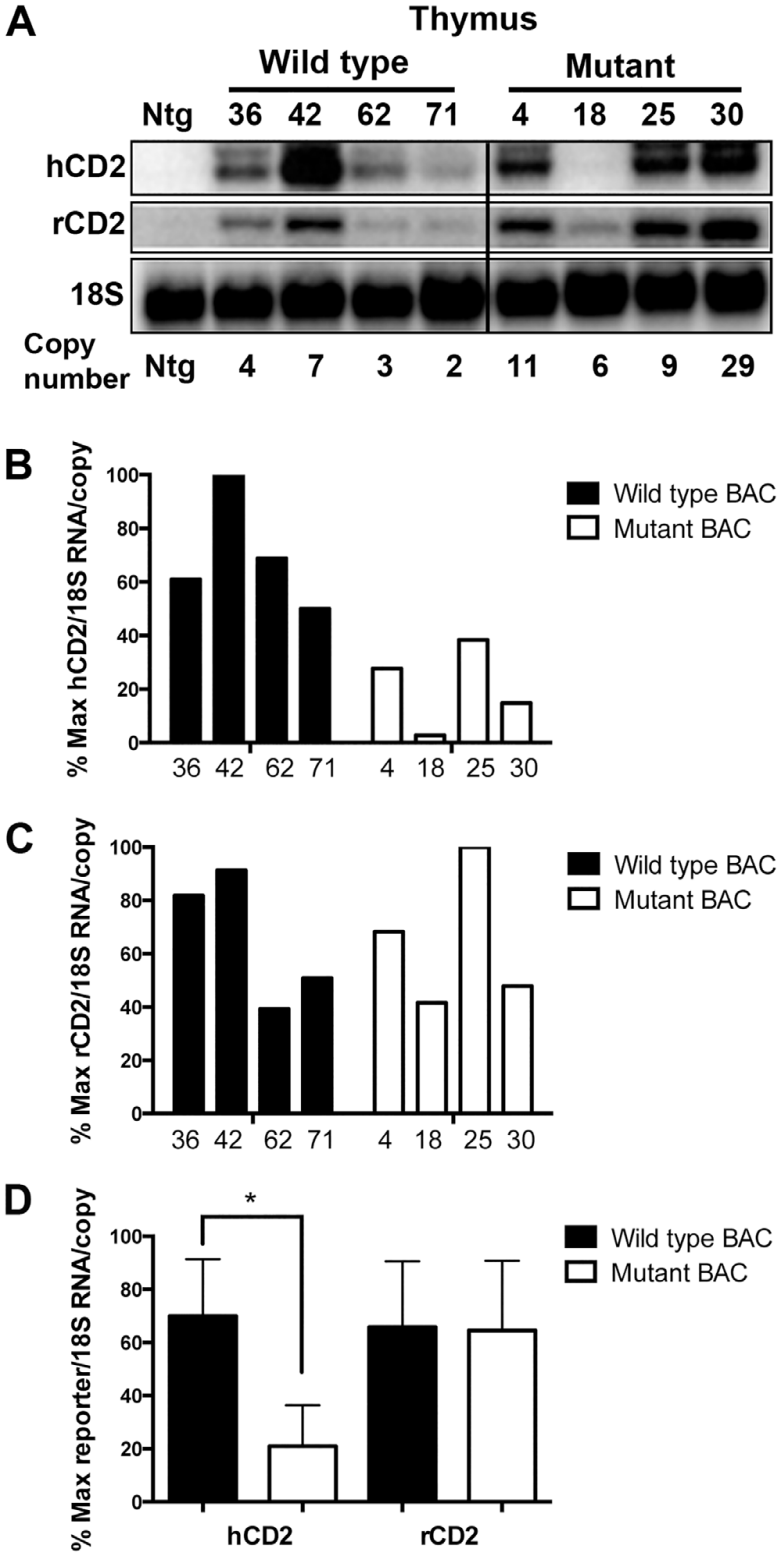
Absence of the deleted region impairs upstream, but not downstream reporter gene expression in thymocytes. **(A)** Northern blot analyses of human and rat CD2 reporter gene mRNA levels in thymocytes from the indicated lines of wild type and mutant reporter BAC transgenic mice. 18S rRNA signals are used as a loading control. Relative transgene copy number for each mouse line is indicated. The black line indicates excision of samples from the blot that are irrelevant to the present study. Panels B and C depict PhosphorImager analyses of the human CD2 **(B)** and rat CD2 **(C)** reporter mRNA signals detected by northern blots. The normalized mRNA levels (per transgene copy) from each wild type (black bars) and mutant (white bars) transgenic mouse line are graphed relative to each other (as % maximum). **(D)** Statistical analyses of the above data using the two-tailed students t test. Graph bars indicate the average (+/- S.E.) normalized mRNA levels among the lines. The asterisk indicates the statistical significance of the difference in hCD2 mRNA levels between wild type and mutant BAC (*p* = 0.012). In contrast, no significant difference in rCD2 mRNA levels was detected (*p* = 0.942).

**Fig 5:**
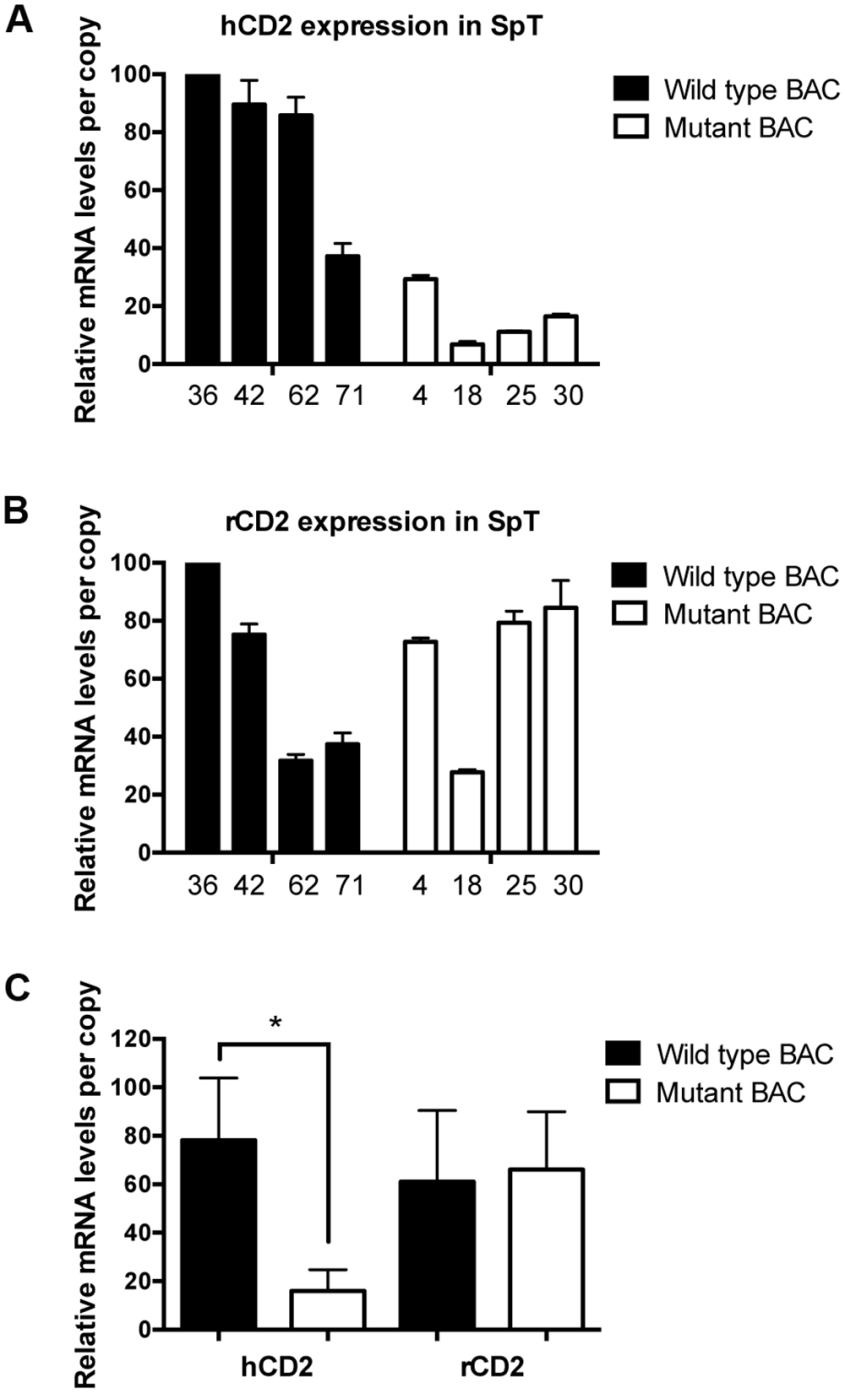
The deleted region is functional in peripheral T cells. qRT-PCR analyses of human **(A)** and rat **(B)** CD2 reporter gene mRNA levels (+/- S.E.) in isolated spleen T cells (SpT) from the indicated lines of wild type (black bars) and mutant (white bars) transgenic mice. Observed reporter mRNA levels per copy from each transgenic line are graphed relative to each other (as % maximum). hCD2 reporter mRNA levels were normalized to endogenous TCRα mRNA levels, and rCD2 expression were normalized to endogenous Dad1 mRNA levels using primers that detect sequences not present in the reporter BAC. Three experiments (S1 Table) were performed in duplicates. **(C)** Statistical analyses of the above data using the two-tailed students t test. The asterisk indicates the statistical significance of the difference in hCD2 mRNA levels between wild type and mutant BAC (p = 0.016). In contrast, no significant difference in rCD2 mRNA levels was detected (p = 0.819). Graph bars indicate the average (+/- S.E.) normalized mRNA levels among the lines.

We next analyzed reporter gene mRNA levels in T cells isolated from wild type and mutant BAC transgenic mice. Northern blot analyses of transgenic thymus RNA samples (Fig 4) indicated that upstream hCD2 reporter mRNA levels (per transgene copy) driven by the mutant reporter BAC were, on average, about one-third of those observed from wild type reporter BAC. In contrast, downstream rCD2 reporter RNA levels were not affected by the mutation. Similar results were obtained from mRNA-level analyses of transgenic peripheral T cells (Fig 5). Per copy levels of hCD2 mRNA driven by the mutant BAC reporter were, on average, nearly five-fold lower than those observed in wild type BAC transgenic T cells. Once again, the levels of rCD2 reporter mRNA per transgene copy were not altered by the deletion mutation. Overall the data demonstrate the function of the deleted region in regulating TCRα-based reporter transgene expression in both thymic and peripheral T cells.

## Discussion

In this report we describe evidence for the presence of a novel *cis*-acting regulatory DNA region in the mouse TCRα gene locus. It is probable that this activity resides within the region of active chromatin we identified between the Jα2 and Cα1 exons. This DNase hypersensitive region of DNA would remain present in the locus after virtually any functional Vα-Jα gene rearrangement event. Thus, this regulatory complex could contribute to TCRα gene expression in the thymus and/or periphery. The residual TCRα gene activity observed in Eα/HS1/HS1’ region knockout mice [7] adds to the rational basis for this notion.

**Fig 6:**
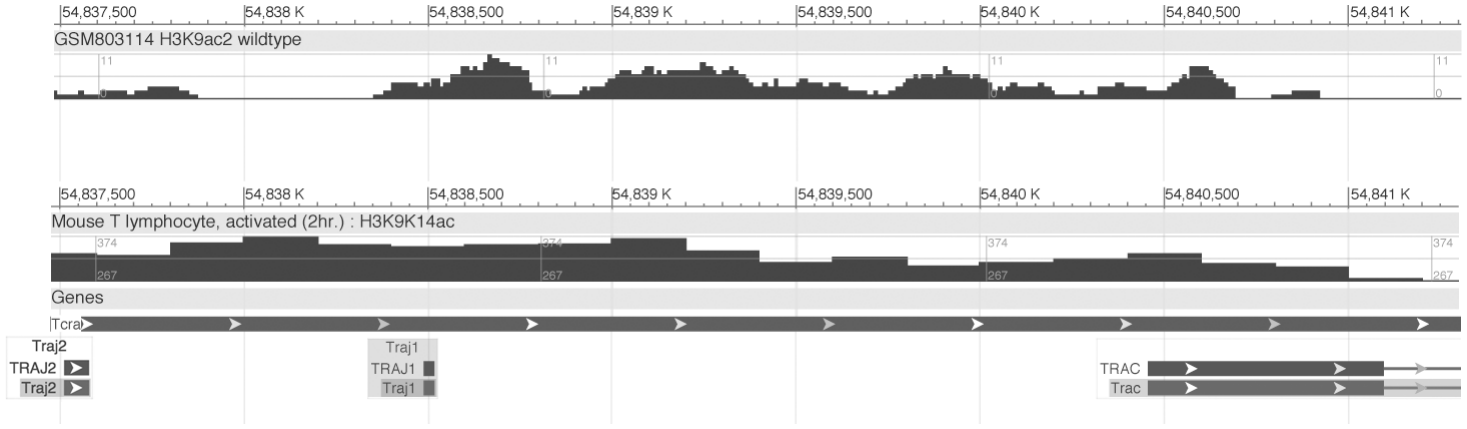
Signatures of active chromatin in the 3’ Jα region. Visualization of histone H3 acetylation marks in CD4^+^,CD8^+^ thymocytes (top track) and activated peripheral CD4^+^ T lymphocytes (bottom track) assayed by ChIP-seq. Shown are screenshots of tracks obtained from publicly available data via the NCBI Epigenomics Browser. The region depicted spans the mouse TCRα gene locus DNA (chromosome 14) containing the Jα2 and Jα1 segments, and the Cα1 constant region exon. Top row: (Unpublished data). ChIP-seq in Mus musculus, strain 129SvJae x C57BL/6 (H3K9ac2). Accession number: ESX000004775. Bottom row: [22]. ChIP-seq in Mus musculus, strain C57BL/6 (H3K9K14ac). Accession number: ESX000001399.

In addition, an early report presented evidence of transcriptional enhancer activity in the Jα-Cα intronic region of the human TCRα gene locus [20]. The enhancer activity described in that report was weaker (~14-fold) than that reported for the 3’ Eα element discovered subsequently in the mouse TCRα locus (>100-fold) [5]. This later report presented evidence suggesting that the corresponding Jα-Cα intronic region of the mouse TCRα locus does not bear significant classical enhancer activity [5]. A contemporaneous study identifying the human counterpart to the 3’-Eα enhancer did not include examination of the human Jα-Cα intron for enhancer activity [21]. In any case, all these prior studies utilized transiently transfected reporter gene bearing plasmids in T cell tumor lines. Here we identified *cis*-acting regulatory DNA 5’ of the Cα exons that is functional in the context of native T cell chromatin in whole mice. Publicly available data from chromatin immunoprecipitation-next generation sequencing (ChIP-Seq) experiments corroborate these findings. The Jα region displays epigenetic signatures of active chromatin, including acetylated histone H3, in T cells. In particular, thymocyte chromatin displays discrete peaks of histone H3 acetylation in the DNA stretching from 5’ of Jα1 into roughly the first third of the Cα1 exon. High histone acetylation levels across this DNA region are also observed in peripheral T cells (Fig 6).

A very recent report provided multiple lines of evidence indicating the inactivity of the Eα element in peripheral T cells [11]. The conclusions of this report are consistent with a prior report indicating that the removal of Eα from a TCRα LCR-driven reporter transgene has virtually no impact on transgene expression in the spleen [6]. Taken together, these findings may help explain why TCRα LCR driven transgenes are expressed at lower levels in peripheral T cells than those observed in thymocytes [4], because the TCRα LCR includes Eα among its functional components [6]. Despite apparent Eα inactivity, it is important to point out that the TCRα LCR, as a whole, remains active in the peripheral T cells, and this finding has been confirmed in combination with four different reporter genes, each bearing its own distinct promoter [4, 12, 15, 23]. Previous reports point to at least two distinct TCRα LCR sub-elements outside of Eα, named HS1’ [6] and HS6 [17, 24], that manifest activity in peripheral lymphoid organs. These elements may also contribute to TCRα mRNA levels in T cell subsets in which the Eα element becomes inactive.

There is strong precedent for antigen receptor gene loci bearing multiple, important *cis*-acting enhancer-like elements located both 5’ and 3’ of their constant region exons. The IgH [25], Igκ [26], TCRγ [27], TCRδ [1] loci all display a version of this arrangement. The findings presented here would add the TCRα gene to this category. The literature has produced a consensus that the multiple *cis*-elements functioning within a particular gene locus can support both redundant and non-overlapping functions (e.g. [27–29]). A subset of these functions can have significant impact on immunity. A recent example of this comes from the IgH gene locus. The intronic Eμ enhancer has long been known to play a key role in V-D-J recombination at the IgH locus [25]. But it was also found to have overlapping function with a 3’ regulatory region complex [30]. Creation of a functional V-D-J knock-in/Eμ knockout IgH allele bypassed Eμ’s role in recombination to enable investigation of its subsequent functions in mice. These studies revealed a surprising impairment of allelic exclusion at the targeted locus in the absence of Eμ [31]. The downstream consequences of this included faulty clonal selection and generation of autoimmune B cell clones [32]. These findings highlight the continuing importance of identifying and studying *cis*-acting elements that might, on the surface, appear either redundant or dispensable for antigen receptor gene function. The multiple genetic and epigenetic processes that occur at these complex gene loci regulate the assembly, timing, level and distribution of the proteins that form the basis of adaptive immunity. The present study adds a new region of the TCRα locus to the collection of potential sources of regulation of these important processes.

## Supporting Information

S1 Table Data from triplicate experiments (Fig 5). (PDF)
