# Supplementary material for: The 3’-Jα Region of the TCRα Locus Bears Gene Regulatory Activity in Thymic and Peripheral T Cells
Source: PLoS One. 2015 Jul 15;10(7):e0132856. doi: 10.1371/journal.pone.0132856 (PMC4503570; doi:10.1371/journal.pone.0132856)
Supplement: S1 Table — (PDF) [file pone.0132856.s001.pdf]

Table S1 – Data from triplicate experiments (Figure 5)

Figure 5A – qRT-PCR Analyses of peripheral T cells (hCD2)

	Normalized hCD2 per copy							
Tg line	Exp 1	Exp 2	Exp 3	Exp 1_ % Max	Exp 2_ % Max	Exp 3_ % Max	Avg_ % Max	S.E.
Wt 36	501.98	392.62	370.42	100	100	100	100	0
Wt 42	487.97	356.22	299.13	97.21	90.73	80.76	89.56	4.78
Wt 62	466.92	320.80	306.46	93.02	81.71	82.73	85.82	3.61
Wt 71	212.31	134.50	130.10	42.29	34.26	35.12	37.22	2.55
Mt 4	154.56	113.54	104.19	30.79	28.92	28.13	29.28	0.79
Mt 18	39.80	23.40	23.70	7.93	5.96	6.40	6.76	0.60
Mt 25	57.46	42.73	41.23	11.45	10.88	11.13	11.15	0.16
Mt 30	84.71	61.03	63.08	16.88	15.54	17.03	16.48	0.47

Figure 5B – qRT-PCR Analyses of peripheral T cells (rCD2)

	Normalized rCD2 per copy							
Tg line	Exp 1	Exp 2	Exp 3	Exp 1_ % Max	Exp 2_ % Max	Exp 3_ % Max	Avg_ % Max	S.E.
Wt 36	96.97	2472.06	492.85	100	100	100	100	0
Wt 42	75.23	1904.43	349.98	77.57	77.04	71.01	75.21	1.49
Wt 62	30.10	843.38	147.85	31.04	34.12	30.00	31.72	0.87
Wt 71	40.60	859.59	175.16	41.87	34.77	35.54	37.39	1.59
Mt 4	71.53	1807.86	351.06	73.77	73.13	71.23	72.71	0.54
Mt 18	26.44	711.90	133.76	27.26	28.80	27.14	27.73	0.38
Mt 25	72.46	2029.73	399.51	74.72	82.11	81.06	79.30	1.63
Mt 30	92.19	2011.45	379.65	95.07	81.37	77.03	84.49	3.84

Figure 5C – qRT-PCR Analyses of peripheral T cells (hCD2 and rCD2)

	n of Tg lines	Avg norm hCD2 RNA	S.E.	p value (two-tailed)	Avg norm rCD2 RNA	S.E.	p value (two-tailed)
Wt	4	78.15	13.97	0.016	61.08	16.17	0.819
Mt	4	15.92	4.88		66.06	13.00	

Tg line: Transgenic mouse line
Wt: Wild type
Mt: Mutant

Avg: Average
Norm: Normalized
S.E.: Standard Error
